# Supplementary material for: In Vivo Ultrafast Doppler Imaging Combined with Confocal Microscopy and Behavioral Approaches to Gain Insight into the Central Expression of Peripheral Neuropathy in Trembler-J Mice
Source: Biology (Basel). 2023 Oct 10;12(10):1324. doi: 10.3390/biology12101324 (PMC10604841; doi:10.3390/biology12101324)
Supplement: Supplementary file 1 [file biology-12-01324-s001.zip › biology-2523831-supplementary.pdf]

Supplementary material

In Vivo Ultrafast Doppler Imaging Combined with Confocal Microscopy and Behavioral Approaches to Gain Insight into the Central Expression of Peripheral Neuropathy in Trembler-J Mice

Mariana Martínez Barreiro [1], Lucia Vázquez Alberdi [1,2], Lucila De León [3], Guadalupe Avellanal [3], Andrea Duarte [1], Maximiliano Anzibar Fialho [2,4], Jérôme Baranger [5], Miguel Calero [6,7], Nicolás Rubido [4,8], Mickael Tanter [5], Carlos Negreira [2], Javier Brum [2], Juan Pablo Damián [3] and Alejandra Kun [1,9,*]

[1] Laboratorio de Biología Celular del Sistema Nervioso Periférico, Departamento de Proteínas y Ácidos Nucleicos, Instituto de Investigaciones Biológicas Clemente Estable, Montevideo 11600, Uruguay; mmartinezb@iibce.edu.uy (M.M.B.); lvazquez@iibce.edu.uy (L.V.A.); aduarte@iibce.edu.uy (A.D.)
[2] Laboratorio de Acústica Ultrasonora, Instituto de Física, Facultad de Ciencias, Universidad de la República, Montevideo 11400, Uruguay; manzibar@fisica.edu.uy (M.A.F.); carlosn@fisica.edu.uy (C.N.); jbrum@fisica.edu.uy (J.B.)
[3] Departamento de Biociencias Veterinarias, Facultad de Veterinaria, Universidad de la República, Montevideo 13000, Uruguay; luciladeleonn@gmail.com (L.D.L.); galloguada@gmail.com (G.A.); jpablodamian@gmail.com (J.P.D.)
[4] Física No Lineal, Instituto de Física de Facultad de Ciencias, Universidad de la República, Montevideo 11400, Uruguay; nicolas.rubidoobrer@abdn.ac.uk
[5] Physics for Medicine Paris, Inserm U1273, ESPCI Paris, PSL University, CNRS UMR 8063, 75012 Paris, France; jerome.baranger@espci.fr (J.B.); mickael.tanter@espci.fr (M.T.)
[6] Unidad de Encefalopatías Espongiformes, UFIEC, CIBERNED, Instituto de Salud Carlos III, 28029 Madrid, Spain; mcalero@isciii.es
[7] Queen Sofia Foundation—Alzheimer Center, CIEN Foundation, 28031 Madrid, Spain
[8] Institute for Complex Systems and Mathematical Biology, University of Aberdeen, King's College, Aberdeen AB24 3UE, UK
[9] Sección Bioquímica, Facultad de Ciencias, Universidad de la República, Montevideo 11400, Uruguay
* Correspondence: akun@fcien.edu.uy; Tel.: +598-2487-1616; Fax: +598-2487-5461

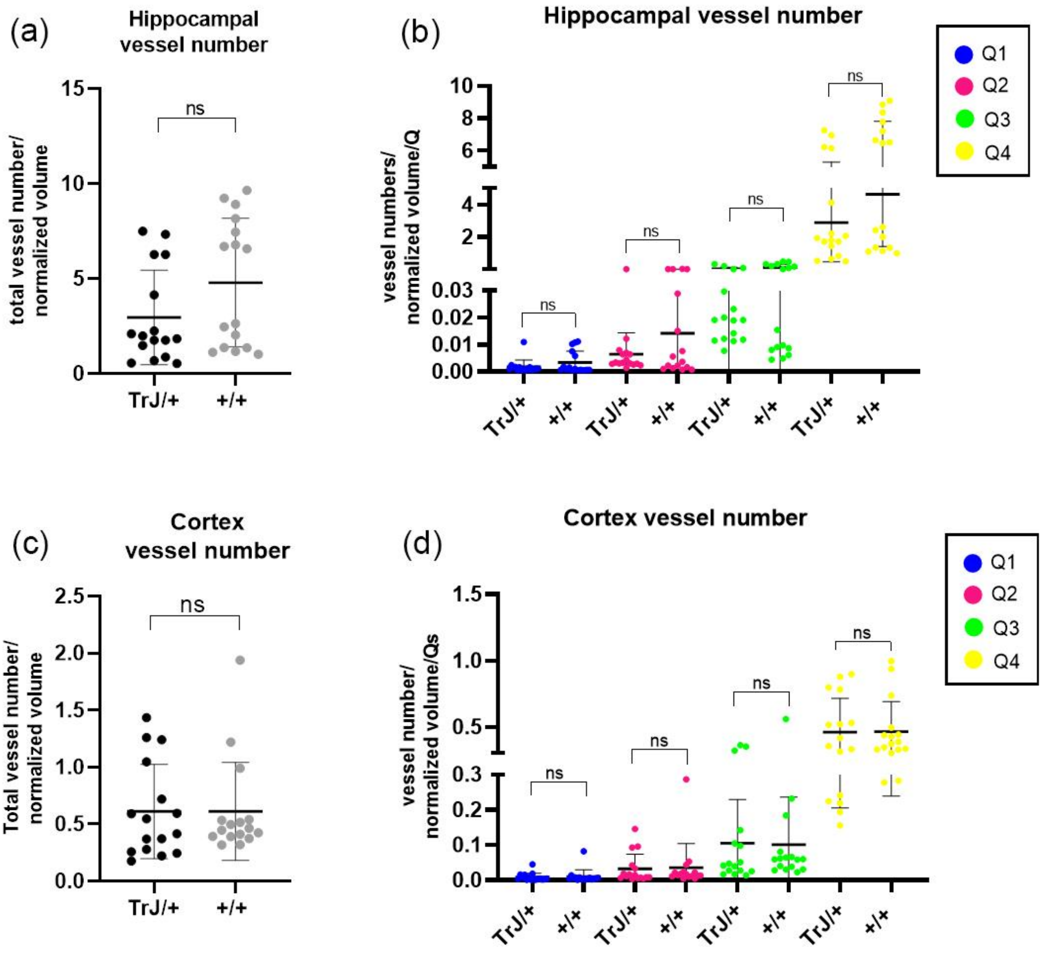

Figure S1: Values of vessel number in the hippocampus and the cortex. (a) Total vessel number in the hippocampus. (b) Total vessel number for each quartile in the hippocampus. (c) Total vessel number in the cortex. (d) Total vessel number for each quartile in the cortex. No significant differences were found in any case.

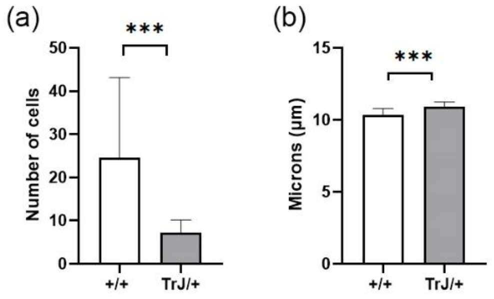

Figure S2: Quantification of erythrocytes in blood samples. (a) Number of erythrocytes in blood. +/+ mice showed a higher concentration of cells than TrJ/+. (b) Diameter of erythrocytes in blood samples. TrJ/+ showed a higher diameter of cells than +/+.
